# 45 Implementing a Multidisciplinary Review Process to Significantly Reduce Peripherally Inserted Central Catheter (PICC) Utilization

**DOI:** 10.1017/ash.2026.10483

**Published:** 2026-06-23

**Authors:** Tomislav Mestrovic

**Affiliations:** 1 University North

## Abstract

**Background:** Antimicrobial resistance (AMR) is increasingly concentrated in hospital settings, where severe infections, older patients and extensive antibiotic exposure converge. While antimicrobial stewardship programs (ASPs) are central to mitigation, long-term global evidence linking resistance patterns to actionable stewardship priorities (and especially for Gram-negative pathogens) has been limited. We assessed three decades (1990–2021) of global AMR trends to identify stewardship-relevant signals for hospital epidemiology and policy, and also developed forecasts scenarios to 2050. **Methods:** We estimated deaths and disability-adjusted life-years (DALYs) attributable to and associated with bacterial AMR for 22 pathogens, 84 pathogen-drug combinations and 11 infectious syndromes across 204 countries from 1990–2021, using the Global Burden of Diseases 2021 framework. Analyses integrated 520 million microbiology, hospital discharge, mortality, pharmaceutical sales and antibiotic use records. AMR burden was quantified under two counterfactuals: replacement of resistant infections with drug-susceptible infections (attributable burden) and replacement with no infection (associated burden). Forecasts to 2050 evaluated a reference scenario, a Gram-negative drug development scenario, and a better-care scenario reflecting improved infection management and access to appropriate antibiotics. **Results:** In 2021, an estimated 4.71 million deaths (95% UI 4.23–5.19) were associated with bacterial AMR, including 1.14 million deaths (1.00–1.28) directly attributable to resistance. Hospital-relevant trends were dominated by Gram-negative resistance: carbapenem-resistant infections showed the largest absolute increase in burden, with associated deaths rising from 0.62 million in 1990 to 1.03 million in 2021. In contrast to declines exceeding 50% among children younger than 5 years, AMR mortality increased by over 80% among adults aged 70 years and older – a population disproportionately affected by bloodstream, respiratory and intra-abdominal infections managed in acute-care settings. By 2050, attributable AMR deaths are projected to reach 1.91 million globally under the reference scenario. Importantly, the better-care scenario (reflecting strengthened stewardship, timely appropriate therapy and improved quality of care) could avert an estimated 92.0 million cumulative deaths between 2025 and 2050, compared with 11.1 million deaths averted under a Gram-negative drug development scenario alone. **Conclusion:** The global AMR burden is increasingly driven by carbapenem-resistant Gram-negative infections in older, hospitalized populations. These findings indicate that antimicrobial stewardship and hospital infection prevention offer substantially greater population-level mortality benefits than reliance on new antibiotic development alone. Policy efforts should prioritize robust ASP implementation, optimization of empiric therapy for severe infections, rapid de-escalation, as well as system-wide improvements in care quality to curb the projected rise in hospital AMR mortality.

